# Gene expression profiling of HCV genotype 3a initial liver fibrosis and cirrhosis patients using microarray

**DOI:** 10.1186/1479-5876-10-41

**Published:** 2012-03-07

**Authors:** Waqar Ahmad, Bushra Ijaz, Sajida Hassan

**Affiliations:** 1 Centre of Excellence in Molecular Biology, University of the Punjab, Lahore, Pakistan; 2 Foreign Faculty Professor HEC, Applied and Functional Genomics Lab, Centre of Excellence in Molecular Biology, University of the Punjab, Lahore, Pakistan

## Abstract

**Background:**

Hepatitis C virus (HCV) causes liver fibrosis that may lead to liver cirrhosis or hepatocellular carcinoma (HCC), and may partially depend on infecting viral genotype. HCV genotype 3a is being more common in Asian population, especially Pakistan; the detail mechanism of infection still needs to be explored. In this study, we investigated and compared the gene expression profile between initial fibrosis stage and cirrhotic 3a genotype patients.

**Methods:**

Gene expression profiling of human liver tissues was performed containing more than 22000 known genes. Using Oparray protocol, preparation and hybridization of slides was carried out and followed by scanning with GeneTAC integrator 4.0 software. Normalization of the data was obtained using MIDAS software and Significant Microarray Analysis (SAM) was performed to obtain differentially expressed candidate genes.

**Results:**

Out of 22000 genes studied, 219 differentially regulated genes found with *P *≤ 0.05 between both groups; 107 among those were up-regulated and 112 were down-regulated. These genes were classified into 31 categories according to their biological functions. The main categories included: apoptosis, immune response, cell signaling, kinase activity, lipid metabolism, protein metabolism, protein modulation, metabolism, vision, cell structure, cytoskeleton, nervous system, protein metabolism, protein modulation, signal transduction, transcriptional regulation and transport activity.

**Conclusion:**

This is the first study on gene expression profiling in patients associated with genotype 3a using microarray analysis. These findings represent a broad portrait of genomic changes in early HCV associated fibrosis and cirrhosis. We hope that identified genes in this study will help in future to act as prognostic and diagnostic markers to differentiate fibrotic patients from cirrhotic ones.

## Background

Chronic hepatitis C is a major liver related health problem destroying liver architecture leading to cirrhosis and hepatocellular carcinoma. Almost 3% of the world population is infected with this deadly virus and in future, it is predicted that infection will rise to 3 fold of the present number [1-6]. HCV persist(s) beside the specific humoral responses and the mechanism of viral persistence and viral clearance is not fully understood. During HCV infection, initial fibrosis development is the method to overcome the damage caused by the virus. But the early events are the basis of disease outcome. Initial fibrosis is thought to be reversible, although many studies do not support this phenomenon. As extracellular matrix (ECM) tissues not only involve matrix production but also matrix degradation leading to ECM remodeling [7-9] Fibrosis is caused by excessive deposition of ECM by histological and molecular reshuffling of various components like collagens, glycoproteins, proteoglycans, matrix proteins and matrix bound growth factors. Fibrosis stage information not only indicates treatment response but also reflect/indicate cirrhosis development disaster [4,10-16]. ECM metabolism is a balance between ECM deposition and removal influenced by cytokines and growth factors [17]. Genome-wide analysis of abnormal gene expression showed transcripts deregulation differences among normal, mild and severe fibrosis during HCC development with identification of novel serum markers for its early stage. Recent studies suggest that genetic markers may be able to define exact stage of liver fibrosis. For this purpose, limited but functional studies have proposed quite a few genetic markers with individual genes or group of genes [18,19]. Advantage of genetic markers over liver biopsy is intrinsic and long-term while, liver biopsy represents only one time point [20]. Researchers found specific genes such as AZIN1, TLR4, CXCL9, CXCL10, CTGF, ITIH1, SERPINF2, TTR, PDGF, TGF-β1, collagens COL1-A1, TNFα, interleukin, ADAMTS, MMPs, TIMPs, LAMB1, LAMC1, Cadherin, CD44, ICAM1, ITGA, APO and CYP2C8 that showed deregulation during liver fibrosis and may be used to access liver fibrosis and cirrhosis [11-28]. Microarray is a powerful technique used for the identification of differentially expressed genes within control and experimental samples in different diseases and conditions like cancer development. Very few studies are available that use microarray for the identification of specific genes related to fibrosis [27,28]. In a recent study, Caillot *et al. *used microarray technique and found a significant association of ITIH1, SERPINF2 and TTR gene expression and their related proteins with all fibrosis stages [28]. Expression of these genes and related proteins gradually decreased during the fibrosis development to its end stage cirrhosis. Mostly, HCV expression based studies using microarray are carried out with genotype 1 and 2. Very few studies exploring the role of HCV genotype 3a are done with limited set of genes using real Time PCR. Those do not represent complete picture of HCV and human gene interaction leading to disease progression [21-28]. In Pakistan, genotype 3a is the major contributor and has strong association with HCC. The aim of the present study was to examine gene expression profiles in the HCV associated liver disease progression. We have identified for the first time, those genes that are differentially regulated in initial fibrosis and advance stage liver cirrhosis 3a patients and identified potential targets that can be used as effective markers to differentiate between fibrotic and cirrhotic liver with genotype 3a. This data may also help to understand the disease stages between initial versus end stage cirrhosis, as there are limited studies concerning HCV genotype 3a disease progression.

## Materials and methods

### Patients

This study was conducted at Department of Pathology, Jinnah Hospital, Lahore, Mayo Hospital, Lahore and Liver Centre Faisalabad with collaboration of Applied and Functional Genomics Lab, National Centre of Excellence in Molecular Biology, University of the Punjab, Lahore, Pakistan. HCV RNA-positive patients were identified among HCV antibody (anti-HCV) positive patients. Patients who had received a previous course of INF or immunosuppressive therapy, or who had clinical evidence of HBV or HIV and any other type of liver cancer were excluded from the study. Patients who refused to have a liver biopsy or for whom it was contraindicated, i.e., because of a low platelet count, prolonged prothrombin time or decompensated cirrhosis were also excluded from the study. The liver biopsy procedure, its advantages and possible adverse effects were explained to the patients. Written informed consent for biopsy procedure was obtained from patients, also contained information about demographic data, possible transmission route of HCV infection, clinical, virological and biochemical data. The study was approved by institutional ethical committee.

### Patients and liver biopsy

A group of patient was selected from previously described study with known fibrosis evaluation [29]. Two groups of samples consisted of early fibrosis (F1) and cirrhosis (F4) containing 9 samples each were made. Patient's characteristics are given in Table 1.

**Table 1:** Clinical Characteristics of the patients used in this study

Factor	Fibrotic patients	Cirrhotic patients	*P *value
Age	37.9 ± 9.5	48.4 ± 7.1	< 0.05
Sex (M/F)	5/4	6/3	0.247
HAI score	6.05 ± 2.8	7.6 ± 2.9	< 0.05
Viral load	1.3 ± × 10^7 ^± 1.5 × 10^7^	2.9 × 10^5 ^± 2.9 × 10^5^	< 0.05
Hb level	12.6 ± 1.2	12.3 ± 1.2	0.328
Bilirubin	0.88 ± 0.2	1.62 ± 0.31	< 0.05
ALT	117.8 ± 55.3	147.5 ± 61.2	0.091
ALP	88.1 ± 47.5	323.8 ± 80.1	< 0.05
AST	107.1 ± 66.5	155.5 ± 90.6	< 0.05
Albumin	4.3 ± 0.16	3.6 ± 0.33	< 0.05
Platelet count	185.1 ± 21.2	81.6 ± 17.7	< 0.05

### RNA isolation, cDNA and aRNA preparation, and dye labeling for microarray experiments

RNA from liver biopsy samples were isolated using RNeasy mini elute kit (Qiagen, USA) and preparation of cDNA and aRNA was carried out using RNA ampulse amplification and labeling kit (Kreatech, USA), according to manufacturer. aRNA from HCV infected patients and normal subjects were labeled with Cy3 and Cy5, respectively. A detailed protocol describing each step from start to microarray hybridization can be downloaded from (http://www.operon.com/products/microarrays/OpArray%20Protocol.pdf).

### Array hybridization and scanning

Biopsy samples were analyzed on cDNA microarrays (Oparray) containing > 22000 named genes with 37584 spots. Equal amount of Cy3 and Cy5 (55 pmol each) labeled targets were mixed with 45 μl of OpArray Hyb Buffer. Pre-washing, array hybridization and post-washing of microarray labeled slides were performed according to the manufacturer protocols at 42°C for 18 hours on fully automated workstation "GeneTAC ™ HybStation".

### Microarray data analysis

GeneTAC ™ UC4 × 4 scanner was used for scanning slides at 10 μm resolution for both Cy3 and Cy5 channels. GeneTAC Integrator 4.0 software was initially used for main data output as "csv" format file containing all necessary information. This "csv" file was converted to "mev" format for normalization by using software "ExpressConverter" (http://www.tm4.org/utilities.html). MIDAS (Microarray Data Analysis System) software was downloaded (http://www.tm4.org/midas.html) and used for normalization of data. Fold induction was determined by using formula log_2_Cy5/Cy3. A rank-based permutation method SAM was used to identify significantly expressed genes among fibrosis stages (http://www-stat.stanford.edu/~tibs/SAM/). Gene expression patterns through k-means clustering were produced and viewed using freely available programs CLUSTER 3.0 (http://rana.lbl.gov/EisenSoftware.htm) and Tree View 1.45 (http://rana.lbl.gov/downloads/TreeView/), respectively. To identify biological themes among gene expression profiles, the Expression Analysis Systematic Explorer (EASE) was used (http://david.abcc.ncifcrf.gov/content.jsp?file=/ease/ease1.htm&type=1) [30]. The microarray data have been deposited to the GEO accession database (http://www.ncbi.nlm.nih.gov/geo) with accession number GSE33258.

### Real-time reverse transcriptase (RT)-PCR analysis

Genes with known function and significantly up-regulated or down-regulated were analyzed by real-time RT-PCR with RNA used for microarray analysis. Total RNA was converted to cDNA using MmLV (Moloney murine leukemia virus). Selected and tested oligonucleotide primer pairs for their specificity were used for real time RT-PCR using ABI 7500 real time PCR system using syber green chemistry. Each experiment was run in triplicate including GAPDH as endogenous control (Table 2). Each gene was quantified relative to the calibrator. Applied Biosystem Sequence Detection Software and calculations were made by instrument using the equation 2^-ΔΔCT^.

**Table 2:** Primer sequences used for Real time RT-PCR analysis

Gene name	Primer sequence	Annealing temp
OAS	s:5'-ACTTTAAAAACCCCATTATTGAAA-3'	58°C
	as:5'-GGAGAGGGGCAGGGATGAAT-3'	
FAM14B	s:5'-TCTCACCTCATCAGCAGTGACCAG-3'	60°C
	as:5'-CCTCTGGAGATGCAGAATTTGG-3'	
CASPASE9	s:5'-ATGTCGTCCAGGGTCTCAAC-3'	58°C
	as:5'-GGAAACTGTGAACGGCTCAT-3'	
TGFBR	s:5'-TTCCGTGGGATACTGAGACA-3'	58°C
	as:5'-AGATTTCGTTGTGGGTTTCC-3'	

## Results

### Patient's characteristics

Among 18 patients, equal number of patients belonged to F1 (9) and cirrhotic (9) group. Out of these, six best samples each with good RNA were used for microarray experiments. Normal liver biopsies were also obtained in triplicate. The serum viral load, bilirubin, albumin, and platelet count of cirrhotic patients were significantly low (*P *< 0.05), while, serum ALP and AST levels were high when compared to patients with F1 stage. There were no significant differences between serum ALT and Hb level in the patients with F1 or cirrhotic stage (Table 1).

### Microarray analysis: expression behavior of significant genes

**Figure 1:**
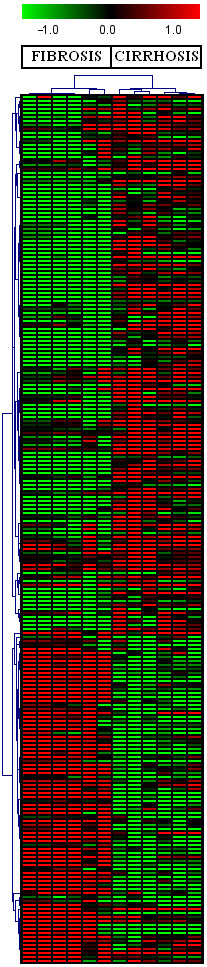
**Significant host genes regulated by HCV infection**. Clustering results for differentially expressed genes between HCV infected patients with initial fibrosis and cirrhosis. Clustering was performed by Cluster 3.0 software. The fold changes in mRNA expression are represented with green and red squares showing down- and up-regulation of genes in liver biopsy samples, respectively. Each vertical column represents an independent experiment, while color scale represents the fold change magnitude.

**Figure 2:**
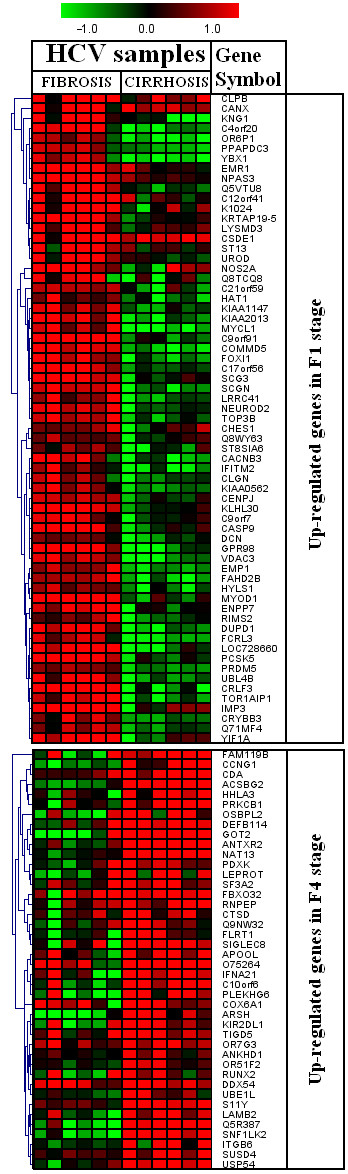
**Heat map of up-regulated genes**. Clustering results for differentially expressed genes between HCV infected patients with initial fibrosis and cirrhosis. Clustering was performed by Cluster 3.0 software. The fold changes in mRNA expression are represented with green and red squares showing down- and up-regulation of genes in liver biopsy samples, respectively. Each vertical column represents an independent experiment, while color scale represents the fold change magnitude.

**Figure 3:**
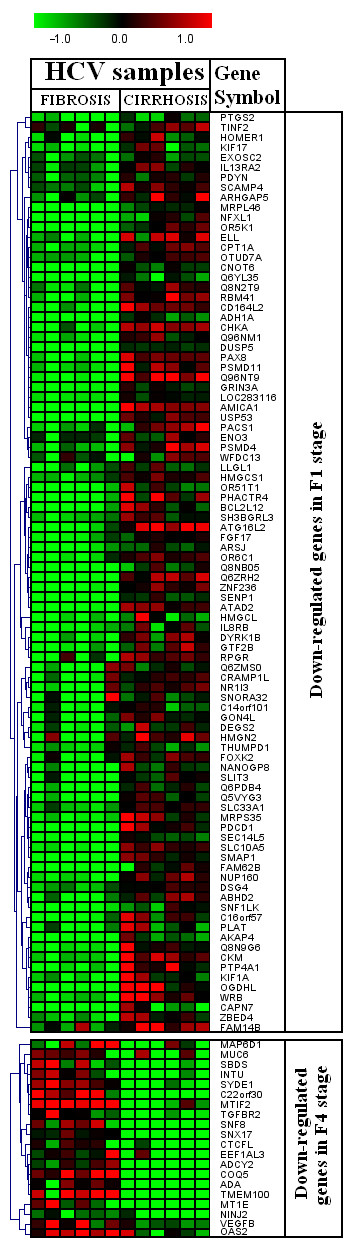
**Heat map of down-regulated genes in cirrhotic and non-cirrhotic sample**. Clustering results for differentially expressed genes between HCV infected patients with initial fibrosis and cirrhosis. Clustering was performed by Cluster 3.0 software. The fold changes in mRNA expression are represented with green and red squares showing down- and up-regulation of genes in liver biopsy samples, respectively. Each vertical column represents an independent experiment, while color scale represents the fold change magnitude.

**Figure 4:**
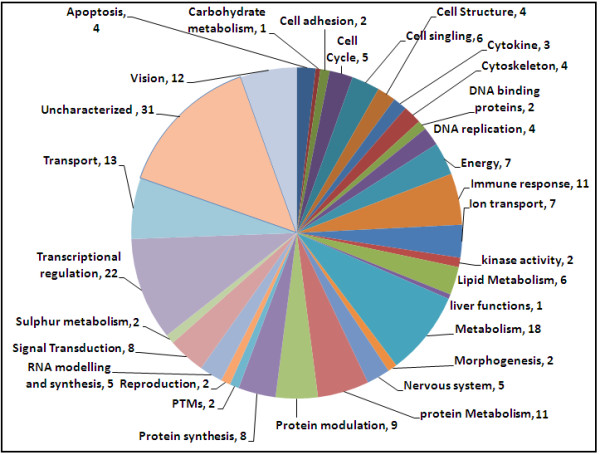
**Distribution of genes according to their functions**. Genes were grouped in 31 different categories.

We found 219 differentially regulated genes in fibrosis versus cirrhotic groups (Figure 1). Among these, 107 genes were up-regulated (Figure 2) whereas, 112 genes were down-regulated (Figure 3). Significant genes with their symbols and functions are listed in Tables 3 and 4. Genes were classified into 31 categories according to their biological functions (Figure 4).

**Table 3:** Up-regulated genes in cirrhotic and non-cirrhotic HCV liver biopsy samples

Function	Symbol	Description	GeneBank	*t*- test
Apoptosis	CASP9	Caspase-9 precursor (EC 3.4.22)	NM_001229.2	0.000207
apoptosis	EMP1	Epithelial membrane protein 1	NM_001423.1	1.38E-06
cell adhesion	YIF1A	Protein YIF1A	NM_020470.1	0.000115
Cell Cycle	CHES1	Checkpoint suppressor 1	NM_005197.2	0.047852
Cell Cycle	CCNG1	Cyclin-G1	NM_004060.3	0.002296
Cell singling	LRRC41	Leucine-rich repeat-containing protein 41	NM_006369.4	0.000132
cell singling	SCG3	Secretogranin-3 precursor	NM_013243.2	4.69E-05
Cell singling	FLRT1	Leucine-rich repeat transmembrane protein FLRT1 precursor	NM_013280.4	0.022495
cell singling	SIGLEC8	Sialic acid-binding Ig-like lectin 8 precursor	NM_014442.2	0.020819
cell structure	HYLS1	hydrolethalus syndrome 1	NM_145014.1	7.28E-05
cell structure	TOR1AIP1	Torsin-1A-interacting protein 1	NM_015602.2	5.13E-05
cytokine	CRLF3	cytokine receptor-like factor 3	XM_001128008.1	3.25E-06
cytokine	PLEKHG6	pleckstrin homology domain containing, family G	NM_018173.1	0.004661
Cytoskeleton	KRTAP19-5	Keratin-associated protein 19-5	NM_181611.1	0.000296
cytoskeleton	COMMD5	COMM domain-containing protein 5	NM_014066.2	8.2E-07
DNA replication	CENPJ	Centromere protein J	NM_018451.2	2.22E-05
DNA replication	TOP3B	DNA topoisomerase 3-beta-1	XM_001129880.1	1.79E-05
DNA replication	DDX54	ATP-dependent RNA helicase	NM_024072.3	0.080445
Energy	Q5VTU8	ATP synthase,	NR_002162.1	9.93E-05
Energy	COX6A1	Cytochrome c oxidase polypeptide VIa-liver	NM_004373.2	0.077561
Immune response	CRYBB3	Beta crystallin B3	NM_004076.3	2.89E-05
Immune response	FCRL3	Fc receptor-like 3 precursor	NM_052939.3	9.14E-06
Immune response	IFITM2	interferon induced transmembrane protein 2	NM_006435.1	0.000197
Immune response	DEFB114	Beta-defensin 114 precursor	NM_001037499.1	0.003039
Immune response	IFNA21	Interferon alpha-21 precursor	NM_002175.1	0.019498
Immune response	KIR2DL1	Killer cell immunoglobulin-like receptor 3DL2 precursor	NM_153443.2	0.014098
Ion transport	CANX	Calnexin precursor	NM_001024649.1	0.097857
Ion transport	CLGN	Calmegin precursor	NM_004362.1	0.002724
ion transport	HHLA3	HERV-H LTR-associating 3 isoform 2	NM_001036645.1	0.003147
kinase activity	PDXK	Pyridoxal kinase	NM_003681.3	0.037672
kinase activity	PRKCB1	Protein kinase C beta type	NM_002738.5	0.009381
Lipid Metabolism	PPAPDC3	Probable lipid phosphate phosphatase PPAPDC3	NM_032728.2	6.06E-05
Lipid Metabolism	OSBPL2	Oxysterol-binding protein-related protein 2	NM_144498.1	0.016902
lipid metabolism	Q5R387	Novel protein	XM_372769.4	0.000376
liver functions	LEPROT	Leptin receptor precursor	NM_017526.2	0.107525
Metabolism	EMR1	EGF-like module-containing mucin-like hormone receptor-like 1 precursor	NM_001974.3	0.00022
Metabolism	UROD	Uroporphyrinogen decarboxylase	NM_000374.3	0.004853
metabolism	DCN	Decorin precursor	NM_001920.3	0.000807
Metabolism	FAHD2B	fumarylacetoacetate hydrolase domain containing 2B	XR_016023.1	4.16E-06
Metabolism	ACSBG2	Prostatic acid phosphatase precursor	NM_001099.2	1.55E-05
Metabolism	ANTXR2	Anthrax toxin receptor 2 precursor	NM_058172.3	1.92E-05
Metabolism	CDA	Cytidine deaminase	NM_001785.2	0.005579
Metabolism	CTSD	Cathepsin D precursor	NM_001909.3	0.028633
Metabolism	GOT2	Aspartate aminotransferase, mitochondrial precursor	XR_016602.1	0.000258
Metabolism	NAT13	Mak3 homolog	XR_018106.1	5.03E-06
Metabolism	TIGD5	Tigger transposable element-derived protein 5	NM_032862.2	0.057722
nervous system	NPAS3	Neuronal PAS domain-containing protein 3	NM_022123.1	0.000115
nervous system	GPR98	G-protein coupled receptor 98 precursor	NM_032119.3	0.000239
nervous system	NEUROD2	Neurogenic differentiation factor 2	NM_006160.3	2.17E-05
nervous system	LAMB2	Laminin subunit beta-2 precursor	NM_002292.3	0.007081
protein Metabolism	CSDE1	GTPase NRas precursor	NM_002524.2	0.017096
protein Metabolism	ENPP7	Ectonucleotide pyrophosphatase	NM_178543.3	0.000321
protein Metabolism	KIAA1147	KIAA1147 (KIAA1147), mRNA	NM_001080392.1	0.000106
protein Metabolism	KIAA2013	KIAA2013 (KIAA2013), mRNA	NM_138346.1	2.86E-05
protein Metabolism	KNG1	Kininogen-1 precursor	NM_000893.2	0.002086
protein Metabolism	APOOL	Protein FAM121A precursor	NM_198450.3	0.019311
Protein modulation	HAT1	Histone acetyltransferase type B catalytic subunit	NM_001033085.1	0.000447
Protein modulation	RIMS2	Regulating synaptic membrane exocytosis protein 2	NM_014677.2	0.000572
Protein modulation	UBL4B	Ubiquitin-like protein 4B	NM_203412.1	2.33E-05
Protein modulation	UBE1L	Ubiquitin-activating enzyme E1 homolog	NM_003335.2	0.021531
Protein modulation	USP54	ubiquitin specific protease 54	NM_152586.2	0.005541
Protein synthesis	RNPEP	Aminopeptidase B	NM_020216.3	0.0218
PTMs	SNF1LK2	Serine/threonine-protein kinase SNF1-like kinase 2	NM_015191.1	0.00037
RNA modelling and synthesis	IMP3	U3 small nucleolar ribonucleoprotein protein IMP3	NM_018285.2	7.13E-05
RNA modelling and synthesis	SF3A2	Splicing factor 3A subunit 2	NM_007165.4	0.123287
Signal Transduction	CACNB3	Voltage-dependent L-type calcium channel subunit beta-3	NM_000725.2	0.00248
Signal Transduction	PCSK5	Proprotein convertase subtilisin/kexin type 5 precursor	NM_006200.2	6.62E-06
Signal Transduction	VDAC3	Voltage-dependent anion-selective channel protein 3	XR_019103.1	0.000231
Signal Transduction	ITGB6	Integrin beta-6 precursor	NM_000888.3	0.008005
sulphur metabolism	FAM119B	family with sequence similarity 119	NM_015433.2	0.018357
Transcriptional regulation	LYSMD3	LysM and putative peptidoglycan-binding domain-containing protein 3	NM_198273.1	0.004237
transcriptional regulation	FOXI1	Forkhead box protein I1	NM_012188.3	1.84E-05
transcriptional regulation	MYCL1	L-myc-1 proto-oncogene protein	NM_001033081.1	4.97E-05
transcriptional regulation	MYOD1	Myoblast determination protein 1	NM_002478.4	7.25E-06
transcriptional regulation	PRDM5	PR domain zinc finger protein 5	NM_018699.2	1.03E-07
Transcriptional regulation	YBX1	Nuclease sensitive element-binding protein 1	XM_001129294.1	6.12E-05
Transcriptional regulation	ANKHD1	Eukaryotic translation initiation factor 4E-binding protein 3	NM_020690.4	0.041134
transcriptional regulation	RUNX2	Runt-related transcription factor 2	NM_001024630.2	0.064668
Transcriptional regulation	SUSD4	Sushi domain-containing protein 4 precursor	NM_017982.2	0.004775
Transport	CLPB	Caseinolytic peptidase B protein homolog	NM_030813.3	0.027308
Transport	K1024	UPF0258 protein KIAA1024	NM_015206.1	0.001564
transport	NOS2A	nitric oxide synthase 2, inducible1	NM_000625	0.017057
transport	SCGN	Secretagogin	NM_006998.3	2E-06
Transport	FBXO32	F-box only protein 32	NM_148177.1	0.043284
Uncharacterized	C12orf41	CDNA FLJ12670	NM_017822.2	0.001604
Uncharacterized	C17orf56	CDNA FLJ31528	NM_144679.1	1.11E-06
Uncharacterized	C21orf59	Uncharacterized protein	NM_021254.1	0.001335
Uncharacterized	C4orf20	CDNA FLJ11200	NM_018359.1	0.000365
Uncharacterized	C9orf7	Uncharacterized protein	NM_017586.1	0.002015
Uncharacterized	C9orf91	C9orf91 protein	NM_153045.2	2.21E-06
Uncharacterized	KIAA0562	glycine-, glutamate-, thienylcyclohexylpiperidine-binding protein	NM_014704.2	5.09E-05
Uncharacterized	KLHL30	kelch-like 30	NM_198582.1	5.06E-06
Uncharacterized	LOC728660	-	XM_001128340.1	0.000153
Uncharacterized	Q71MF4	-	-	7.89E-05
Uncharacterized	Q8TCQ8	CDNA FLJ90801 fis, clone Y79AA1000207	XM_001134000.1	0.028312
Uncharacterized	Q8WY63	PP565	-	0.017959
Uncharacterized	ST8SIA6	Alpha-2,8-sialyltransferase 8F	NM_001004470.1	0.008458
Uncharacterized	C10orf6	Uncharacterized protein C10orf6	NM_018121.2	0.000673
Uncharacterized	O75264	-	XM_209196.5	0.01282
Uncharacterized	Q9NW32	CDNA FLJ10346	-	0.038301
Uncharacterized	S11Y	Putative S100 calcium-binding protein	XM_001126350.1	0.002549
Vision	ST13	Hsc70-interacting protein	XR_018201.1	0.012047
Vision	DUPD1	dual specificity phosphatase and pro isomerase domain containing 1	NM_001003892.1	4.5E-06
Vision	OR6P1	Olfactory receptor 6P1	-	1.78E-05
Vision	ARSH	arylsulfatase H	NM_001011719.1	0.002229
Vision	OR51F2	Olfactory receptor 51F2	NM_001004753.1	0.018938
Vision	OR7G3	Olfactory receptor 7G3	NM_001001958.1	0.077667

**Table 4:** Down-regulated genes in cirrhotic and non-cirrhotic HCV liver biopsy samples

Function	Symbol	Description	GeneBank	*t*- test
Apoptosis	BCL2L12	Bcl-2-related proline-rich protein	NM_001040668.1	0.000335
Apoptosis	PDCD1	Programmed cell death protein 1 precursor	NM_005018.1	7.94E-06
carbohydrate metabolism	OGDHL	oxoglutarate dehydrogenase-like	NM_018245.1	0.000909
cell adhesion	THUMPD1	THUMP domain-containing protein 1	NM_017736.3	0.044141
Cell Cycle	AKAP4	A-kinase anchor protein 11	NM_016248.2	0.000598
cell cycle	TINF2	TERF1-interacting nuclear factor 2	NM_012461	0.045359
cell cycle	VEGFB	vascular endothelial growth factor B	NM_003377	0.017886
cell singling	GRIN3A	Glutamate [NMDA] receptor subunit 3A precursor	NM_133445.1	1.17E-06
cell singling	Q8N9G6	similar to nuclear pore membrane protein 121	XM_498333.2	7.46E-05
Cell Structure	ENO3	Beta-enolase	NM_001976.2	0.050309
cell structure	MAP6D1	MAP6 domain-containing protein 1	NM_024871.1	0.034981
cytokine	IL13RA2	Interleukin-13 receptor alpha-2 chain precursor	NM_000640.2	0.024814
Cytoskeleton	LLGL1	Lethal(2) giant larvae protein homolog 1	NM_004140.3	0.008177
cytoskeleton	SNX17	Sorting nexin-17	NM_014748.2	5.41E-05
DNA binding proteins	ZNF236	Zinc finger protein 236	NM_007345.2	1.54E-07
DNA binding proteins	ZBED4	Zinc finger BED domain-containing protein 4	NM_014838.1	0.003728
DNA replication	WRB	Tryptophan-rich protein	NM_004627.2	0.000578
Energy	ABHD2	ATP-binding cassette sub-family F member 2	NM_005692.3	0.000193
Energy	ATAD2	ATPase family AAA domain-containing protein 2	NM_014109.2	8.59E-06
Energy	PSMD11	26S proteasome non-ATPase regulatory subunit 11	NM_002815.2	0.000415
Energy	PSMD4	26S proteasome non-ATPase regulatory subunit 4	NM_002810.2	0.003878
Energy	SYDE1	synapse defective 1	NM_033025.4	0.000222
Immune response	ATG16L2	ATG16 autophagy related 16-like 2	NM_033388.1	1.31E-05
Immune response	IL8RB	High affinity interleukin-8 receptor B	NM_001557.2	0.00539
immune response	PTGS2	prostaglandin-endoperoxide synthase 2	NM_000963	0.00504
immune response	FAM14B	Interferon alpha-inducible protein 27-like protein 1	NM_145249	0.006008
immune response	OAS2	2'-5'-oligoadenylate synthase 2	NM_016817	0.009299
Ion transport	DSG4	Desmoglein-4 precursor	NM_177986.2	1.23E-05
Ion transport	SLC10A5	Sodium/bile acid cotransporter 5 precursor	NM_001010893.2	6.5E-08
Ion transport	CAPN7	Calpain-7	NM_014296.2	0.003785
ion transport	MT1E	Metallothionein-1E	NM_175617.3	0.001767
Lipid Metabolism	DEGS2	sphingolipid C4-hydroxylase/delta 4-desaturase	NM_206918.1	0.008105
lipid metabolism	CHKA	Choline kinase alpha	NM_001277.2	0.003609
lipid metabolism	ADA	bubblegum related protein	NM_030924.3	6.6E-05
Metabolism	HMGCL	Hydroxymethylglutaryl-CoA lyase, mitochondrial precursor	NM_000191.2	0.010122
metabolism	HMGCS1	Hydroxymethylglutaryl-CoA synthase, cytoplasmic	NM_002130.4	1.2E-05
Metabolism	SH3BGRL3	SH3 domain-binding glutamic acid-rich-like protein 3	NM_031286.3	0.000157
Metabolism	ARHGAP5	Rho GTPase-activating protein 5	NM_001173.2	0.010513
Metabolism	CKM	Creatine kinase M-type	NM_001824.2	0.000689
Metabolism	CPT1A	Carnitine O-palmitoyltransferase I, liver isoform	NM_001031847.1	0.008256
Metabolism	USP53	Inactive ubiquitin carboxyl-terminal hydrolase 53	NM_019050.1	2.46E-06
morphogenesis	SLC33A1	Acetyl-coenzyme A transporter 1	NM_004733.2	8.12E-06
morphogenesis	PDYN	Beta-neoendorphin-dynorphin precursor	NM_024411.2	0.055803
nervous system	NINJ2	Ninjurin-2 (Nerve injury-induced protein 2)	NM_016533.4	0.004163
protein Metabolism	GON4L	GON-4-like protein	NM_001037533.1	0.00137
protein Metabolism	PHACTR4	phosphatase and actin regulator 4 isoform 1	NM_001048183.1	0.000139
protein Metabolism	OTUD7A	OTU domain-containing protein 7A	XM_001127986.1	0.00394
protein Metabolism	Q96NT9	GR AF-1 specific protein phosphatase	XM_497354.1	7.5E-05
protein Metabolism	WFDC13	Protein WFDC13 precursor	NM_172005.1	0.080737
Protein modulation	SMAP1	Stromal membrane-associated protein 1	NM_001044305.1	8.97E-08
Protein modulation	DYRK1B	Dual specificity tyrosine-phosphorylation-regulated kinase 1B	NM_004714.1	0.001009
Protein modulation	COQ5	Ubiquinone biosynthesis methyltransferase COQ5	NM_032314.3	2.07E-05
Protein modulation	MTIF2	Translation initiation factor IF-2	NM_001005369.1	0.002917
Protein synthesis	MRPL46	39S ribosomal protein L46, mitochondrial precursor	NM_022163.2	2.83E-06
Protein synthesis	MRPS35	28S ribosomal protein S35, mitochondrial precursor	NM_021821.2	0.000245
protein synthesis	PLAT	Tissue-type plasminogen activator precursor	NM_000930.2	0.014382
protein synthesis	SENP1	Sentrin-specific protease 1	NM_014554.2	1.63E-06
protein synthesis	ELL	RNA polymerase II elongation factor ELL	NM_006532.2	0.003242
Protein synthesis	PACS1	Phosphofurin acidic cluster sorting protein 1	NM_018026.2	0.005118
protein synthesis	PTP4A1	Protein tyrosine phosphatase type IVA protein 1	NM_003463.3	0.001949
PTMs	SNF1LK	Serine/threonine-protein kinase SNF1-like kinase 1	NM_173354.3	0.000169
Reproduction	LOC283116	similar to Tripartite motif protein 49	XR_016154.1	5.32E-07
Reproduction	Q5VYG3	OTTHUMP00000018545	-	2.51E-05
RNA modelling and synthesis	EXOSC2	Exosome complex exonuclease RRP4	NM_014285.4	0.080373
RNA modelling and synthesis	RBM41	RNA-binding protein 41	NM_018301.2	0.002623
RNA modelling and synthesis	ADCY2	Double-stranded RNA-specific adenosine deaminase	NM_001111.3	6.64E-05
Signal Transduction	FGF17	Fibroblast growth factor 17 precursor	NM_003867.2	0.000254
Signal Transduction	ADH1A	Adenylate cyclase type 2	NM_020546.2	0.00139
Signal Transduction	HOMER1	Homer protein homolog 1	NM_004272.3	0.011954
Signal Transduction	TMEM100	Transmembrane protein 100	NM_018286.1	3.31E-05
sulphur metabolism	FAM62B	family with sequence similarity 62	NM_020728.1	1.6E-05
transcriptional regulation	CRAMP1L	Protein cramped-like	NM_020825.2	0.006587
transcriptional regulation	FOXK2	Forkhead box protein K2	XM_001134364.1	0.00156
transcriptional regulation	HMGN2	Nonhistone chromosomal protein HMG-17	XM_001133530.1	0.01162
Transcriptional regulation	NANOGP8	Homeobox protein NANOGP8	-	0.000264
transcriptional regulation	NFXL1	nuclear transcription factor	NM_152995.4	8.53E-05
transcriptional regulation	NR1I3	Orphan nuclear receptor NR1I3	NM_001077470.1	0.00247
Transcriptional regulation	SNORA32	Protein JOSD3	NR_003032.1	0.107321
Transcriptional regulation	GTF2B	Transcription initiation factor IIB	NM_001514.3	0.002357
Transcriptional regulation	PAX8	Paired box protein Pax-8	NM_003466.3	4.89E-05
Transcriptional regulation	CTCFL	Transcriptional repressor CTCFL	NM_080618.2	0.003129
Transcriptional regulation	EEF1AL3	Eukaryotic translation elongation factor 1 alpha 1	-	0.000917
Transcriptional regulation	INTU	PDZ domain-containing protein 6	NM_015693.2	0.003842
transcriptional regulation	TGFBR2	TGF-beta receptor type-2 precursor	NM_001024847.1	0.007651
Transport	KIF1A	Kinesin-like protein KIF1A	NM_004321.4	0.002119
transport	NUP160	Nuclear pore complex protein Nup160	NM_015231.1	3.11E-06
transport	SLIT3	Slit homolog 3 protein precursor	NM_003062.1	0.000577
Transport	AMICA1	Junctional adhesion molecule-like precursor	NM_153206.1	3.86E-06
Transport	KIF17	Kinesin-like protein KIF17	NM_020816.1	0.007279
Transport	SCAMP4	secretory carrier membrane protein 4	NM_079834.2	0.026864
transport	MUC6	Mucin-6 precursor (Gastric mucin-6)	XM_290540.7	0.054436
Transport	SNF8	Vacuolar sorting protein SNF8	XR_019363.1	0.000425
Uncharacterized	C14orf101	Uncharacterized protein C14orf101	NM_017799.3	0.02931
Uncharacterized	C16orf57	C16orf57 protein	NM_024598.2	0.000456
Uncharacterized	Q6PDB4	-	-	2.68E-05
Uncharacterized	Q6ZMS0	CDNA FLJ16729	-	0.027141
Uncharacterized	Q6ZRH2	CDNA FLJ46361	-	1.15E-06
Uncharacterized	Q8NB05	CDNA FLJ34424	-	0.000459
Uncharacterized	SEC14L5	-	XM_032693.5	2.77E-06
Uncharacterized	CD164L2	CD164 sialomucin-like 2 protein precursor	NM_207397.2	0.000576
Uncharacterized	CNOT6	CCR4-NOT transcription complex subunit	NM_015455.3	0.000838
Uncharacterized	Q6YL35	-	-	0.00218
Uncharacterized	Q8N2T9	CDNA: FLJ21438	XM_029084.8	0.007508
Uncharacterized	Q96NM1	CDNA FLJ30594	-	0.000447
Uncharacterized	C22orf30	Novel protein (DJ694E4.2 protein)	NM_173566.1	0.000611
Uncharacterized	SBDS	Shwachman-Bodian-Diamond syndrome	NM_016038.2	0.006543
Vision	ARSJ	arylsulfatase family, member J	NM_024590.3	0.000249
vision	OR51T1	Olfactory receptor 51T1	NM_001004759.1	0.000408
vision	OR6C1	Olfactory receptor 6C1	NM_001005182.1	0.000136
vision	DUSP5	Dual specificity protein phosphatase 5	NM_004419.3	0.000125
vision	OR5K1	Olfactory receptor 5K1 (HTPCRX10)	NM_001004736.2	0.000169
Vision	RPGR	retinitis pigmentosa GTPase regulator	NM_001023582.1	0.007569

### Significantly synchronized genes with known biological functions

The differentially regulated genes were grouped according to their biological functions by EASE program that uses information from Entrez Gene (http://jura.wi.mit.edu/entrez_gene/) and KEGG database (http://www.genome.jp/kegg/kegg1.html). Our results showed variation in gene regulation in both early fibrosis and cirrhosis stages (Figure 1). Out of 107 up-regulated gens, 65 belonged to early fibrosis stage, whereas, 42 genes belonged to the cirrhotic stage. Genes related to immune response, cell signaling, kinase activity, lipid metabolism, metabolism, vision and transcriptional regulation were up-regulated in both early fibrosis and cirrhotic samples (Table 2). We found that most genes related to apoptosis, cell structure, cytoskeleton, nervous system protein metabolism, protein modulation, signal transduction, transcriptional regulation and transport were up-regulated in early fibrosis. Many uncharacterized genes were also found up-regulated in liver disease progression. We identified 112 genes (F1 = 92; F4 = 20) related to above mentioned pathways down-regulated when fibrosis lead to cirrhotic stage (Table 2 and Figure 2). Genes related to these pathways showed varied response and none of biological function was specifically related to any liver disease stage (Table 4 and Figure 3).

### Independent validation of candidate genes using quantitative real-time RT-PCR

Total RNA extracted from infected liver biopsies was used for real time RT-PCR analysis to validate microarray data. Expression analysis of the genes involved in apoptosis, immune response and transcriptional regulation was performed. We randomly selected four genes, CASPASE9, FAM14B, OAS2 and TGFBR2 from our study. CASPASE9 is apoptosis related gene, FAM14B and OAS2 are immune responsive genes, whereas, TGFBR2 is multifunctional gene and found to be up-regulated in fibrosis.

## Discussion

Liver fibrosis can progress to cirrhosis after an interval of 15-20 years in patients with HCV [31]. It is very important to identify such markers that can differentiate liver fibrosis from cirrhosis. Liver biopsy is a common tool for the detection of liver current situation but due to some limitations its use as diagnostic tool is denied. Microarray analysis is an emerging and novel approach to study gene expression in HCV associated fibrosis and cirrhosis. As liver gene expression in HCV patients is variable and it might be partially dependent on the corresponding genotype [32]. In this study, we specially focused on gene expression analysis in patients with genotype 3a that is most common in our region. We found that many genes associated with apoptosis, several cellular functions, immune response, metabolism including energy, liver, sulphur; protein metabolism, transcriptional regulation, signal transduction, transport, DNA replication were dys-regulated both in early fibrosis and cirrhosis. In some cases, gene expression tends to be increased from initial fibrosis to cirrhosis. Induction of gene expression associated with proapoptotic, proinflammatory and proliferative activities is in accordance with previous studies [18,27,33-35]. Although, we found some dysregulation of genes related to vision and nervous system first time.

### Differential expression of apoptosis related genes in HCV associated initial fibrosis and cirrhosis

**Figure 5:**
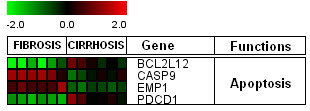
**Differential expression of apoptotic genes in HCV associated initial fibrosis and cirrhosis. **Clustering results for differentially expressed genes between HCV infected patients with initial fibrosis and cirrhosis according to their functions. Clustering was performed by Cluster 3.0 software. Genes shown in red are up-regulated, while down-regulated genes are shown in green. Genes shown in black have no expression changes. Gene expression profiles were presented on a 2-fold change scale.

In this study, host genes involved in apoptosis (Figure 5) such as BCL212 and PDCD1 showed down-regulation in initial fibrosis and significant up-regulation in cirrhosis, whereas, expression levels for CASP9 and EMP1 genes were high at initial stage and were down-regulated in cirrhosis stage. Regulation of apoptotic inducer and program cell death genes, BCL212 and PDCD1 in cirrhosis is according to previous observations where pro-apoptotic gene signaling has been observed in infection with HCV [36,37]. CASP9 is known as apoptosis initiator [38] and EMP1 is also found to induce apoptosis [39,40]. Expression of caspases is higher in early and moderate HCV infection, and enhanced apoptosis occur through the intrinsic apoptotic pathway via mitochondria [41,42].

### Cellular functions, cell cycle, signaling and cytoskeleton associated genes

**Figure 6:**
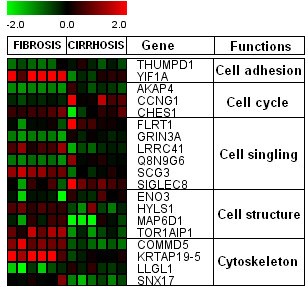
**Parallel expression of genes associated with Cellular functions, cell cycle, signaling and cytoskeleton in F1 versus F4. **Genes shown in red are up-regulated, while down-regulated genes are shown in green. Genes shown in black have no expression changes. Gene expression profiles were presented on a 2-fold change scale.

Genes related to various cellular functions showed different expression patterns (Figure 6). The cytoskeleton (COMMD5, KRTAP19-5, LLGL1 and SNX17) related genes were down-regulated in cirrhosis (F4). Most cell structure related genes were up-regulated in initial fibrosis (HYLS1, MAP6D1 and TOR1AIP1) and genes related to cell adhesion, cell cycle and signaling showed differential expression in both initial fibrosis and cirrhosis. It has been observed that HCV RNA synthesis may require an intact cytoskeleton [43]; our data indicated that many genes related to cytoskeleton were regulated by HCV infection.

### Genes associated with Immune response and cytokines

**Figure 7:**
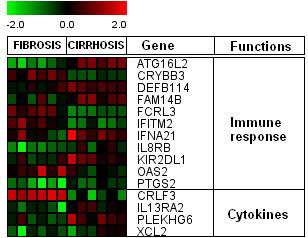
**Expression profiles of immune responsive and cytokines associated genes. **Clustering results for differentially expressed genes between HCV infected patients with initial fibrosis and cirrhosis according to their functions. Clustering was performed by Cluster 3.0 software. Gene expression profiles were presented on a 2-fold change scale.

A number of genes related to immune response and cytokines were identified (Figure 7). ATG16L2, DEFB114, FAM14B, IFNA21, IL8RB and KIR2DL genes were up-regulated in cirrhosis, whereas, FCRL3, IFITM2 and OAS2 genes were up-regulated in initial fibrosis. Genes related to cytokine regulation, IL13RA2, PLEKHG6 and XCL2 were down-regulated in initial fibrosis except CRLF3 gene. Interleukin related gene expression has been found to be increased at pathology stage 3 and 4 and which is concurrent with the present study and is associated with metastatsis, cell proliferation or angiogenesis [37,44]. An increased expression of immune responsive genes and cytokines as fibrosis progress is in agreement with previous evidence that liver inflammation may enhance with increase in infected hepatocytes [45]. FCRL3, a genetically conserved gene family encodes orphan cell surface receptors bearing high structural homology to classical Fc receptors, with multiple extracellular Ig domains and either ITAMs, ITIMs, or both in the intracellular domains. The natural ligands of these family members are still unknown but due to their signaling domains and expression on multiple immune cell types, these members likely modulate immune cell functions by affecting signaling pathways [46]. FCRL3 is expressed predominantly in B lymphocytes in lymph nodes and germinal centers [47-49].

Previous studies revealed that IFITM2 and IFITM3 (two structurally related cell plasma membrane proteins) interrupt early steps entry and/or uncoating of the viral infection. Interferon-induced transmembrane (*IFITM*) genes are transcribed in most tissues with the exception of *IFITM5 *interferon inducible gene. IFITM genes are involved in early development, cell adhesion, and control of cell growth. Elevated gene expression triggered by past or chronic inflammation can prevent spreading of pathogens by limiting host cell proliferation. Low level of expression is sufficient to capture the growth of cells, whereas, the loss of expression causes tumor growth. This gene is termed as tumor suppressor. However, in many cancers it is observed that despite high level of IFITM, it represents tumor progression stage especially where the one of anti-proliferative interferon pathway is shut down. The role of ATG protein in membrane trafficking is mostly not clear. ATGL16 is thought to play role in autophagosome formation in association with RAB33B. It is also considered an active player in HCV replication and assembly [50,51].

Natural killer cells are the important player of innate immune response. KIRDL gene expression is found to be high in chronic HCV patients [52]. We found the KIR2DL1 gene expression high in patients with cirrhosis as compared to initial fibrosis stage. OAS synthesized in response to IFN-alpha stimulation. In infected cells, OAS enzymatic activity is induced by double-stranded RNAs, such as the intermediates of replication of RNA viruses or folded single stranded RNAs. OAS catalyzes polymerization of adenosine triphosphate into oligoadenylate that, in turn, activates a cellular endoribonuclease, RNase L, at subnanomolar concentrations. RNase L degrades cellular and viral single-stranded RNAs. Thus, viral replication is inhibited as a result of protein synthesis inhibition in a totally non-virus specific way [53]. We found high expression of OAS2 gene in fibrotic samples as compared to the last stage cirrohsis. This may be a way to stop viral replication but as the disease steps forward, virus overcome the host immune response to replicate itself.

### Genes associated with different metabolic processes

**Figure 8:**
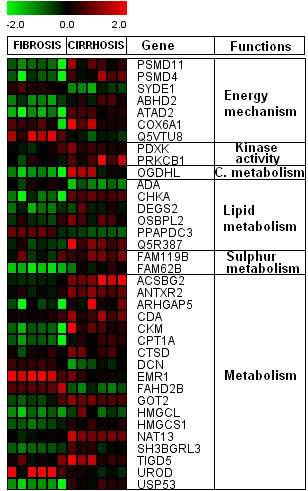
**Genes associated with different metabolic processes**. Clustering was performed by Cluster 3.0 software. Genes shown in red are up-regulated, while down-regulated genes are shown in green. Genes shown in black have no expression changes. Gene expression profiles were presented on a 2-fold change scale.

A number of genes associated with different metabolism (processes/pathways) like energy, kinases, lipid and sulphur metabolism were identified among significantly expressed arrays (Figure 8). Several studies observed that HCV induces alterations in lipid metabolism that can lead to oxidative stress [54,55]. Consistent with these observations, we found six genes, ADA, CHKA, DEGS2, OSBPL2, PPAPDC3, and Q5R387; which are involved in lipid biosynthesis, tumor cell growth by phosphatidyl-ethanolamine biosynthesis, negative regulation of myoblast differentiation and hydrolyzation of phospholipids into fatty acids etc. This finding is in agreement with Diamond *et al*.; that host cell lipid metabolism may represent an area for future HCV antiviral therapies [56]. We found two genes FAM119B and FAM62B associated with sulphur metabolism which were up-regulated in cirrhotic samples. A number of genes related to energy mechanism such as PSMD4, PSMD11, ABHD2, ATAD2 and COX6A1 were up-regulated while, SYDE1 and Q5VTUB genes were down-regulated in cirrhotic samples. Two genes PDXK and PRKCB1 with kinase activity, and one gene, OGDHL linked to carbohydrate metabolism were also identified. Role of PRKCB1 (also known as PKC) in cell growth and differentiation control is known. It has been also found elevated in breast and pituitary tumors and malignant gliomas [57-59]. PKC was also found up regulated in hepatocellular carcinoma which can lead to hyper proliferation of the HCV infected tissues [60].

### Genes associated with protein synthesis, modulation and metabolism

**Figure 9:**
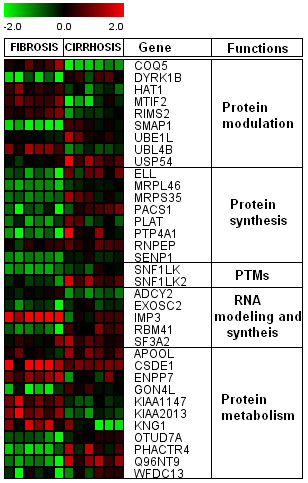
**Genes associated with protein synthesis, modulation and metabolism**. Genes shown in red are up-regulated, while down-regulated genes are shown in green. Genes shown in black have no expression changes.

Many genes involved in protein synthesis, modulation and metabolism have increased or decreased expression in patients with HCV (Figure 9). Genes representing protein synthesis were down-regulated in initial fibrosis and showed significant increased expression in cirrhotic samples. Two genes associated with protein post-translational modifications (PTMs) were also identified that showed increased expression in cirrhosis. Some genes linked with protein metabolism like GON4L, OTUD7A, PHACTR4, Q96NT9 and WFDC13 showed low expression in initial fibrosis, while CSDE1, ENPP7, KIAA1147, KIAA2013 and KNG1 were up-regulated in early fibrosis. It was interesting to know that previous studies have not shown the regulation of PTMs and protein synthesis with respect to HCV, although other viruses such as HIV have shown these trends. However, our findings were in agreement with Blackham et al. who showed these types of regulations in HCV infected hepatocytes [61].

### Transcriptional regulation and signal transduction related genes

**Figure 10:**
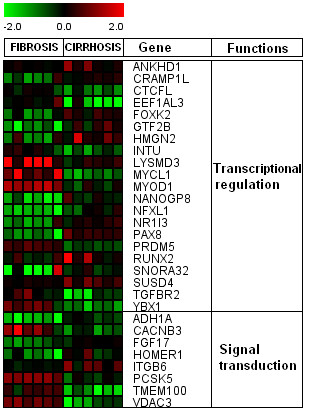
**Expression of transcription and signal transduction related genes.** Clustering was performed by Cluster 3.0 software.

Several genes associated with transcriptional regulation and signal transductions were identified (Figure 10). Most genes were down-regulated both in HCV initial fibrosis and cirrhosis. However, ANKHD1, CRAMP1L, FOXK2, GTF2B, HMGN2, NR1I3, PAX8, RUNX2 and SUSD4 genes showed increased trend in cirrhotic samples. Xu *et al. *also reported up-regulation of liver enriched transcriptional factors in infected HCV tissues [62]. A comprehensive study is needed to address the exact role of these genes. Some genes associated with signal transduction like CACNB3, PCSK5, TMEM100 and VDAC3 were up-regulated in initial fibrosis. Up-regulation of signal transduction related genes in HCC due to HCV and HBV is previously reported [63,64]. This can lead to the hypothesis that cirrhosis due to HCV genotype 3a may lead to HCC in future.

### Transport and ion channel transport related genes

**Figure 11:**
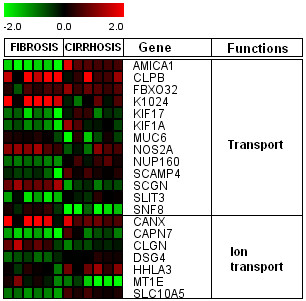
**Regulation of transport and ion channel related genes by HCV. **Clustering results for differentially expressed genes between HCV infected patients with initial fibrosis and cirrhosis according to their functions. Genes shown in red are up-regulated, while down-regulated genes are shown in green. Genes shown in black have no expression changes.

A number of genes encoding cellular and ion transport functions were also recognized (Figure 11). AMICA1, HHLA3, KIF17, KIF1A and SLC10A5 showed significant high expression, while, CLPB, K1024, MUC6, SCGN and MT1E expression was down in cirrhotic arrays. Previous studies related to HCV infection and entry has shown that HCV replication needs regulations in cellular trafficking [65-67]. High expression of SLC10A5, also known as putative bile acid transporter gene, it may indicate dysregulation of liver as well as pancreas in patients infected with HCV. Up-regulation of kinesin family members KIF17 or KIF2B may upset inner segment and synaptic terminal and consequently results in cell death [68].

### Others significant genes

**Figure 12:**
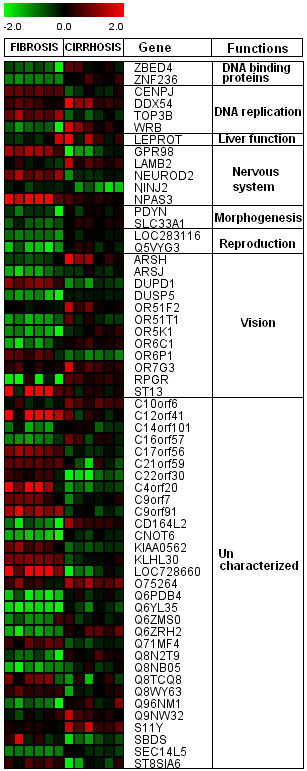
**Differential expression of genes associated with DNA binding, DNA replication, liver function, nervous system, vision and uncharacterized.** Clustering results for differentially expressed genes between HCV infected patients with initial fibrosis and cirrhosis according to their functions. Clustering was performed by Cluster 3.0 software. Genes shown in red are up-regulated, while down-regulated genes are shown in green. Genes shown in black have no expression changes. Gene expression profiles were presented on a 2-fold change scale.

Irrespective of above mentioned genes; we have also found several genes related to DNA binding proteins, DNA replication, morphogenesis, reproduction and liver function (Figure 12). The expression of DNA binding protein and replication genes change from initial fibrosis to cirrhosis. The high expression in early fibrosis may underlie a repair mechanism, whereas, reduced gene expression in cirrhosis stage may indicate that virus has overcome the repair mechanism for its replication resulting in total deterioration of liver cells and structure. It is interesting to note that some genes associated with nervous system and vision pathways were also identified. A lot of uncharacterized genes were also recognized. The link of expression of vision related genes with HCV is not clear.

### Real time RT-PCR validation of results

**Figure 13:**
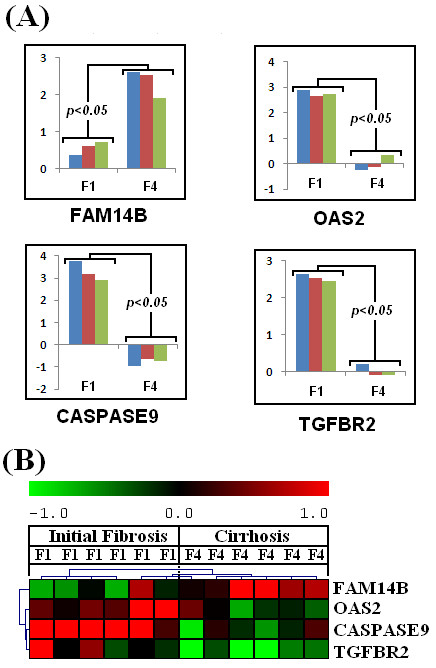
**Validation of microarray data by RT-PCR**. (**A**) Quantification of differential expression of randomly selected genes by real time RT-PCR. (**B**) Expression profile of selected genes from our microarray study.

Analysis with real time RT-PCR confirmed that the selected genes were significantly differentially expressed in initial fibrosis and cirrhotic samples (Figure 13). Although, we observed higher fold induction values with real time RT-PCR, however, the trend was same between both analysis indicating reproducible gene expression patterns. CASPASE9, OAS2 and TGFBR2 genes showed up-regulation, whereas, FAM14B gene expression was down-regulated in early fibrosis. These findings open a new spectrum of genetic markers to differentiate fibrosis from cirrhosis.

A comprehensive review of literature revealed that very few studies related to HCV expression based studies leading to initial to final stage cirrhosis have been carried out in association to genotype. Walters *et al. *used J6/JFH (genotype 2a) infected Huh-7.5 cells for the expression analysis of host in response to virus at different time points of infection. They observed that TGF-beta signaling genes were up-regulated 72 hrs post infection, it induces ROS activity. Liver injury during chronic HCV infection is immune mediated [37]. Hagist *et al. *compared differentially expressed genes in patients with mild and severe iron depleted HCV genotype 1a liver samples with hereditary hemochromatosis. They found many ISG genes dysregulated in HCV infection and related to RNA processing and carcinogenesis [69]. We also found up-regulation of ISG genes in initial fibrosis stage as host defense system try to limit the viral pathogenesis. A study conducted by Blackham *et al. *in JFH1 infected huh-7 cells by microarray identified genes mainly apoptosis, proliferation, intracellular transport and cellular mechanism [61]. A few studies to explore the role of individual genes of HCV in pathogenesis have been studied in association to genotype. Shah *et al. *compared the expression of oxidative stress related genes in blood samples and found that the expression of COX-2, iNOs and VEGF was high in 3a in comparison to 1a [70]. We found the expression is high in initial fibrosis stage and down regulation at the advance stage of liver cirrhosis.

## Conclusion

There are limited studies available dealing with gene expression profiling in cirrhotic and non-cirrhotic (initial fibrosis) patients infected with HCV. In this study, we have observed that HCV infection due to genotype 3a has widespread effects on host gene expression involved in apoptosis, metabolism, transport, transcriptional regulation and immune response. This gives comprehensive information about the pathogenesis caused by HCV genotype 3a leading from initial to end stage liver cirrhosis. Although, HCV genotype 3a showed same pathways activation caused by other genotypes, further studies are required to understand the mechanism by which different genotypes can affect various pathways. Meanwhile, we found that expression of these genes was significantly changed within initial and final stage of fibrosis. A study describing the progression of these genes in mild and severe fibrosis stages (F2 and F3) will be required for future perspectives.

## Competing interests

The authors declare that they have no competing interests.

## Authors' contributions

WA and BI contributed equally to this work. They analyzed the data and wrote paper. All work was performed under supervision of SH. We all authors read and approved the final manuscript.

## Authors' information

WA and BI are research officers at CEMB, while SH (PhD Molecular Biology) is Principal Investigator at CEMB, University of the Punjab, Lahore.
